# Considerations When Assessing Endurance in Combat Sport Athletes

**DOI:** 10.3389/fphys.2019.00205

**Published:** 2019-03-05

**Authors:** Oliver R. Barley, Dale W. Chapman, Stuart N. Guppy, Chris R. Abbiss

**Affiliations:** ^1^Centre for Exercise and Sports Science Research, School of Medical and Health Sciences, Edith Cowan University, Joondalup, WA, Australia; ^2^Performance Support, New South Wales Institute of Sport, Sydney, NSW, Australia

**Keywords:** physiological assessment, performance monitoring, measurement precision, biology of combat sports, sports specificity, aerobic capacity

## Abstract

Combat sports encompass a range of sports, each involving physical combat between participants. Such sports are unique, with competitive success influenced by a diverse range of physical characteristics. Effectively identifying and evaluating each characteristic is essential for athletes and support staff alike. Previous research investigating the relationship between combat sports performance and measures of strength and power is robust. However, research investigating the relationship between combat sports performance and assessments of endurance is less conclusive. As a physical characteristic, endurance is complex and influenced by multiple factors including mechanical efficiency, maximal aerobic capacity, metabolic thresholds, and anaerobic capacities. To assess endurance of combat sports athletes, previous research has employed methods ranging from incremental exercise tests to circuits involving sports-specific techniques. These tests range in their ability to discern various physiological attributes or performance characteristics, with varying levels of accuracy and ecological validity. In fact, it is unclear how various physiological attributes influence combat sport endurance performance. Further, the sensitivity of sports specific skills in performance based tests is also unclear. When developing or utilizing tests to better understand an athletes’ combat sports-specific endurance characteristic, it is important to consider what information the test will and will not provide. Additionally, it is important to determine which combination of performance and physiological assessments will provide the most comprehensive picture. Strengthening the understanding of assessing combat sport-specific endurance as a physiological process and as a performance metric will improve the quality of future research and help support staff effectively monitor their athlete’s characteristics.

## Introduction

Combat sport is a term used to describe a wide range of competitive contact sports typically involving physical combat where the winner is determined by specific criteria depending on the rules of the sport. Combat sports have a large public following with sports such as boxing and mixed martial arts (MMA) having millions of followers and approximately 20% of summer Olympic medals available in combat sports such as boxing, judo and taekwondo (Franchini et al., 2012; James et al., 2016b; Reale et al., 2016). Combat sports can be categorized as grappling, striking or mixed style sports. Grappling sports involving gripping, throwing, ground combat, chokeholds and joint locks (Ratamess, 2011), while striking sports incorporate skills ranging from only punches to combinations of punches, kicks, knees, and elbows (Rodrigues Silva et al., 2011). Mixed style combat sports involve both grappling and striking, thus requiring a diverse skill-set (Tack, 2013). The rules vary between combat sports resulting in different methods of victory and competition durations, which in turn results in many possible competitive styles, even within the same sport (Tack, 2013). In several combat sports, specific techniques or positions are worth a set number of points and successfully applied techniques are then subsequently totalled to determine victory should an opponent not be defeated via a submission, knock-out (KO) or technical knock-out (TKO; Balmer et al., 2005; Ratamess, 2011). In contrast, some sports utilize an ever-evolving and more subjective scoring system for bouts that are not ended by a submission, KO or TKO. For example, the current scoring system applied to both amateur and professional bouts in several combat sports is referred to as the “10-Point Must System,” whereby judges subjectively decide the winner of the round awarding 10 points and the opponent 9 or less. These scoring systems result in diverse methods of victory in combat sports, and thus the term “performance” is complicated. Indeed, overcoming an opponent through submission in the first minute of an event or on points after fifteen minutes of fighting would both be examples of successful competitive performances but achieved through very different physical characteristics, skill sets and tactics. Thus, for coaches and physical conditioning support personnel assessing such physical characteristics is important for optimizing athlete development, competition tactics and preparations for competition. As a result, a large body of literature has been developed around understanding the physical and physiological characteristics of combat sport athletes (Chaabene et al., 2018; James et al., 2016b).

Combat sports are physically demanding, requiring a diverse physical and physiological profile to be successful in competition (Kraemer et al., 2001; Ratamess, 2011; Bridge et al., 2014; Franchini et al., 2014; Chaabène et al., 2015). Striking movements such as punches and kicks require explosive strength and power (Loturco et al., 2014; House and Cowan, 2015), while grappling movements can require a greater emphasis on isometric and concentric strength (Ratamess, 2011; James et al., 2016b). Additionally, combat sports are comprised of many different sports-specific movements which will influence the physical load. For instance, sports such as boxing and judo exert a greater demand on the upper limbs whilst taekwondo exerts a greater demand on the lower limbs (Bridge et al., 2014; Franchini et al., 2014; Chaabène et al., 2015). Even differences in equipment requirements may influence the physical demands of the sport, such as the use of a kimono in Brazilian Jiu-Jitsu and judo increasing the use of the forearm muscles (Andreato and Branco, 2016). The specific skills and rulesets of a combat sport will significantly influence the energy cost of competition (Crisafulli et al., 2009; Andreato and Branco, 2016; Hausen et al., 2017). However, combat sports do not typically involve a single execution of one particular technique but instead involve repeated executions interspersed with lower intensity actions (Rodrigues Silva et al., 2011; Franchini et al., 2013; Andreato et al., 2015; Miarka et al., 2015a,b). The high-intensity repeat-effort nature of combat sports typically results in a large aerobic response during exercise as demonstrated by athletes reaching near maximal heart rates and oxygen consumption (>90% of maximum HR and VO_2max_) during simulated competition (Crisafulli et al., 2009; Doria et al., 2009; Campos et al., 2012). Additionally, the high-intensity component of combat sport competitions induces significant anaerobic strain with research observing high levels of blood lactate (>12 mmol.L^-1^) following competition (Bouhlel et al., 2006; Hanon et al., 2015). This makes endurance an important characteristic of success in competitive combat sports (Amtmann and Berry, 2003; La Bounty et al., 2011; Ratamess, 2011; Lenetsky and Harris, 2012). However, endurance is a difficult concept to define as it is influenced by a wide range of physiological, psychological and biomechanical factors (Abbiss and Laursen, 2005). For the purposes of this review endurance will defined as the ability to maintain a high-intensity or repeated efforts over longer exercise durations. The work:rest ratios of high-intensity efforts during competition vary, thus resulting in different endurance profiles between combat sports (Del Vecchio et al., 2011; Rodrigues Silva et al., 2011; Andreato et al., 2015). It is also important to consider the total duration of events in combat sports, with some sports having a single round lasting 10 min and others involving up to twelve 3 min rounds. Furthermore, it is possible that different regulations in performance enhancing drugs and weight cutting practices may also influence performance in different combat sports and levels of competition. These differences create complications when attempting to compare results acquired from exercise testing of athletes from different types of combat sports, especially Olympic and professional ones (Andreato and Branco, 2016). Additionally, the rules of many combat sports are somewhat unique and allow for an athlete to win before the allotted competition time, which makes the total duration highly variable. Indeed, it has been reported that over half of all MMA fights in the Ultimate Fighting Championship^®^ (UFC^®^) are ended within the first round (Del Vecchio and Franchini, 2013). However, it is not uncommon for fights to last the entire allocated duration (Miarka et al., 2015b). These diverse requirements, due to both the physical requirements and varied length of competition, result in athletes requiring several well-developed physical characteristics including strength, power, agility and endurance on top of technical skill and tactics to be successful (Amtmann and Berry, 2003; La Bounty et al., 2011; Ratamess, 2011). While there are a wide range of potential tools for support staff to use to assess an athlete’s relevant physical characteristics, what approaches are most relevant to combat sports success currently are not clear, especially in the case of combat sports endurance related performance (Chaabene et al., 2018). As such, the purpose of this review is to provide a critical appraisal of the current methods of assessing physical capacities in combat sports with a focus on endurance ability in an effort to help develop best practice guidelines.

## Current Practices in AssessingPhysical Characteristics inCombat Sports

It may not be possible to develop a single test that simultaneously assesses all factors of all combat sports performance under controlled conditions. As a result, the best available approach is to assess individually characteristics integral to competitive success, such as strength, power, agility and endurance (La Bounty et al., 2011; Ratamess, 2011; James et al., 2016b) with some insight into the underlying physiology to lesser or greater degree. Effectively identifying and evaluating such characteristics is essential to inform training interventions, nutritional demands, talent identification, and design the optimal competition strategies for athletes. When selecting a test to assess a characteristic in combat sports athletes, it is important to differentiate between tests where the primary outcome measure is an indicator of physiological capacity or performance. Tests of performance are primarily used to simulate competition in a controlled manner as opposed to assessing the function of a physiological system (Bassett and Howley, 2000; Currell and Jeukendrup, 2008). When evaluating the body of research investigating key physical characteristics in combat sports, it is apparent that the employed methods range from physiological assessments with low sports specificity to performance assessments with high sports specificity (Franchini et al., 2011; Chaabène et al., 2012; James et al., 2016b). Thus assessments seeking to isolate an underlying physical characteristic with low sports specificity used across combat sports include: one repetition maximum (1RM) testing (Franchini et al., 2007; Garthe et al., 2011; James et al., 2016b), maximal isokinetic strength assessment (Timpmann et al., 2008), counter-movement and squat jumps (Fogelholm et al., 1993; Garthe et al., 2011; Ouergui et al., 2014; James et al., 2016b; Barley et al., 2018b), 40-m sprint (Garthe et al., 2011), 30 s continuous jump (Čular et al., 2018), repeated contractions on an isokinetic dynamometer (Moore et al., 1992; Kraemer et al., 2001; Oopik et al., 2002; Timpmann et al., 2008; Barley et al., 2018a), Wingate testing (Fogelholm et al., 1993; Artioli et al., 2010; Mendes et al., 2013; Durkalec-Michalski et al., 2014; Ouergui et al., 2014), various repeat-sprint tests (Barley et al., 2018b; James et al., 2018) and maximal aerobic capacity testing (Guidetti et al., 2002; Ravier et al., 2006; Franchini et al., 2011; Bruzas et al., 2014; Reljic et al., 2015; James et al., 2016b). As the spectrum of physical assessments shifts toward a higher level of sport specificity assessment, examples include exercise circuits (i.e., burpees, press-ups, and sports-specific skills such as throws or strikes) (Smith et al., 2000; Franchini et al., 2005, 2007, 2011; Hall and Lane, 2001; Artioli et al., 2010; Chaabène et al., 2012; Villar et al., 2016; Sant’Ana et al., 2017) and simulated combat with a live opponent (Yang et al., 2018) (Table 1). Although the use of such testing modalities appears sound, critical evaluation of the ecological validity of the test or physiological characteristic involved is required, including an understanding of the precision to detect small but important changes.

**Table 1:** Common methods to assessment physical capacities in combat sports athletes.

Assessment	Physical capacity assessed	Reference within combat sports	Does the assessment involve sports-specific skills	Primary outcome variable	Lower body or upper body engaged	Repeat effort	Combat sport the test can be used for
One repetition maximum testing	Strength	Franchini et al., 2007	No	Weight lifted	Upper or lower body	No	Generic
Maximal isokinetic strength assessment	Strength	Barley et al., 2018a	No	Torque generated	Upper or lower body	No	Generic
Counter-movement and squat jumps	Power	Barley et al., 2018a	No	Jump height, and force generated	Lower body	No	Generic
30-s continuous jump	Anaerobic power and capacity	Čular et al., 2018	No	Number of jumps and height of jumps	Lower body	Yes	Generic
Repeated contractions on an isokinetic dynamometer	Repeat-effort endurance	Barley et al., 2018a	No	Torque generated and number of contractions	Upper or lower body	Yes	Generic (depending on effort-relief intervals)
Wingate anaerobic assessment	Power and anaerobic capacity	Ouergui et al., 2014	No	Peak power, average power and fatigue index	Upper or lower body	No (repeated Wingate protocols can be designed)	Generic
Maximal aerobic capacity testing	Aerobic capacity and continuous effort endurance ability	Bruzas et al., 2014	Possibly (in most cases no)	Maximal oxygen consumption and workload achieved	Upper, lower or whole body	Depends on the protocol	Longer duration combat sports (i.e., multiple rounds)
Special Judo fitness test	Repeat-effort endurance and anaerobic capacity	Franchini et al., 2011	Yes	Index (calculated by heart rate and number of throws)	Whole body	Yes	Judo
Karate-specific aerobic test	Repeat-effort endurance and aerobic capacity	Chaabène et al., 2012	Yes	Time to exhaustion	Whole body	Yes	Karate
Repeat sled-push test	Repeat-effort endurance	Barley et al., 2018b	No	Average run speed, total test time and peak sprint test	Whole body	Yes	Mixed martial arts
Repeated sprint ability test	Repeat-effort endurance	James et al., 2018	No	Mean sprint time	Whole body	Yes	Mixed martial arts (effort-relief interval adjustments could make the test apply to other sports)
Taekwondo anaerobic test	Anaerobic power and capacity	Sant’Ana et al., 2014	Yes	Number of repetitions, test time and kick force	Whole body	Yes	Taekwondo
Specific jiu-jitsu anaerobic performance test	Repeat-effort endurance and anaerobic capacity	Villar et al., 2016	Yes	Number of repetitions	Whole body	Yes	Jiu-jitsu


There is a belief that when assessing a performance characteristic, the assessment should relate as closely as possible to the sport itself. However, there can be a trade-off between precision of measurement for the physical characteristic and maintaining sporting relevance. While some assessment methods of physical characteristics closely relate to competitive success (James et al., 2016a,b), the relationship for others is much less clear (James et al., 2016b). For example, greater levels of strength and power have been linked to a higher competitive level in combat sports (James et al., 2016a,b) and to greater punching force (Loturco et al., 2014; House and Cowan, 2015). In fact, it appears that the body of research assessing strength and power in combat sports athletes is robust (Loturco et al., 2014; House and Cowan, 2015; Iermakov et al., 2016; James et al., 2016a,b). In contrast, the methods of assessing combat sports-specific endurance are highly varied in the literature and much less robust (Chaabene et al., 2018). This is likely due to the complex nature of endurance as a physical characteristic underpinning combat sport performance, making it far more complicated to assess. Indeed, it is acknowledged that the demand on the aerobic system varies depending on intensity and competition length, with sports involving multiple rounds such as boxing, kickboxing and MMA placing a greater strain on the aerobic system (Smith, 1998; La Bounty et al., 2011; Rodrigues Silva et al., 2011; Alm and Yu, 2013; Del Vecchio and Franchini, 2013; Chaabène et al., 2015). Additionally, sports with single rounds likely require a significant aerobic contribution (Chaabène et al., 2012; Ratamess, 2011; Franchini et al., 2014). However, it is important to note that endurance is influenced by many more factors than just aerobic capacity (Coyle, 1999; Bassett and Howley, 2000; Aziz et al., 2007; Buchheit, 2008; Aguiar et al., 2016). The repeated high-intensity efforts involved require competitive athletes to have well developed strength-endurance, efficiency and anaerobic capacities alongside a capacity to rapidly recover (Coyle, 1999; Ratamess, 2011; Bridge et al., 2014; Chaabène et al., 2015; Salci, 2015). Thus, to better understand endurance ability in a combat sport athlete it is important to consider all factors relevant to the individual combat sport’s-specific endurance. Given the lack of clarity in the literature regarding the assessment of endurance relevant to combat sports, the following sections seek to provide recommendations for methods of evaluating characteristics relevant to endurance ability in combat sports athletes.

## Assessing Endurance in Combat Sports Athletes From a Performance Perspective

When assessing endurance performance, the overall duration and intermittent nature of the specific combat sport should be taken into account (Del Vecchio and Franchini, 2013). Combat sports with multiple rounds can involve greater than 30 min of high-intensity intermittent activity (Guidetti et al., 2002; Andreato and Branco, 2016). Additionally, combat sport bouts involve the use of a wide range of sports-specific skills that can also influence the physical requirements which may be difficult to replicate under controlled conditions (Bridge et al., 2014; Franchini et al., 2014; Chaabène et al., 2015; Andreato and Branco, 2016). Whilst it is generally understood that utilizing sports-specific assessments is ideal (Müller et al., 2000), developing a sports-specific and scientifically valid assessment of endurance for all combat sport athletes is difficult. This is due to the high degree of variation in the physiological demands, sports-specific skills and competitive approaches both between and within combat sports (Franchini et al., 2011; Chaabène et al., 2012).

The variation between and within combat sports complicates the assessment of endurance in such athletes. As a result, researchers examining endurance capacity in combat sport athletes have utilized a range of assessment methods. These include circuits of activities conducted in a manner that reflect the required physical and physiological load of the specific sport, and in many cases, including sports-specific skills such as strikes and throws to mimic the performance aspects required (Smith et al., 2000; Hall and Lane, 2001; Franchini et al., 2005, 2007, 2011; Artioli et al., 2010; Chaabène et al., 2012; Villar et al., 2016; Sant’Ana et al., 2017) (Table 1). In locomotion sports such as cycling or running, the goal of any assessment is to evaluate such locomotion as this is the context in which competitive performance occurs. But in sports such as combat sports, where locomotion is not the sole objective, there are many goals and measures of possible performance. Specifically, the ability to continually attack and defend effectively against an opponent during later rounds is essential to victory (Miarka et al., 2015b). The ability to continue to execute sports-specific skills over longer periods of time despite fatigue has been assessed in judo (Franchini et al., 2011), karate (Chaabène et al., 2012), boxing (Smith et al., 2000), taekwondo (Sant’Ana et al., 2014) and Brazilian jiu-jitsu (Villar et al., 2016) (Table 1). These tests all involve the repeated execution of one or more sports-specific skill for an allocated time or until volitional fatigue, with varying measurements recorded. Simulation-style tests such as these provide valuable information on the fatigue induced by such sports-specific drills. There remain however many combat sports that do not have sports-specific performance tests available, and future research should aim to address this gap in available methodology. While it is important to consider that the potential limitations of such testing methods have not been completely explored, these include aspects related to the precision of *in situ* aerobic capacity measurement, lactate and ventilatory threshold identification and more general instances of measurement and repeatability of test performance. The special judo fitness test has undergone reliability testing as well as physiological examination (Franchini et al., 2009, 2011). Indeed, ventilatory gas analysis identified that 28.2 ± 2.9% of energy requirement were aerobic during this test (Franchini et al., 2011). An issue with this and many other performance based tests is that they are unable to assess important physiological characteristics such as thresholds, efficiency of motion or others likely to be relevant to performance. Regardless, typical tests of repeat-ability are derived from locomotion tasks, such as repeated-sprint ability, where the relationship between fatigue and changes in biomechanics is better understood (Morin et al., 2006). However, in combat sports the changes in technical ability resulting from fatigue and how important such changes are to competitive success are not clear. Given the highly technical nature of combat sports it is plausible that fatigue-induced reductions in skill would have an even greater impact on competitive success than in locomotion based sports. Current sports-specific protocols do not comprehensively monitor impairments in skill which could result in missing important information that would plausibly have a substantial influence on performance during real competition. To better understand this future research should investigate the specific kinematic changes in combat sports techniques resulting from fatigue.

Repeat-effort ability is regarded as essential in a wide range of sports outside of those centered on combat. As a result there is a substantial body of literature that examines the repeat-effort ability of athletes (Bishop et al., 2001). While assessments of repeat-effort ability will have a similar basic structure, they can vary in a range of ways, including the duration of efforts, the recovery duration and number provided, and the modality of exercise (Bishop et al., 2001; Aziz et al., 2007). Previously reported testing protocols have involved repeated sprints on foot (Zagatto et al., 2009; James et al., 2018), on a cycle ergometer (Bishop et al., 2001), upper-body ergometer (Mendes et al., 2013), or pushing a sled (Barley et al., 2018b). Generic running tests such as the 30–15 intermittent fitness test are common examples of repeat-effort running tests commonly used in the field (Aziz et al., 2000; Buchheit, 2008; James et al., 2018), although the work-rest ratios are unlikely to be reflective of combat sports competitions. Many repeat-effort assessments however, only measure the high-intensity component (i.e., the sprint duration) while neglecting the low-intensity recovery portion, which potentially results in missing important information (Balsom et al., 1992; Spencer et al., 2005; Barley et al., 2018b; James et al., 2018). For example, when assessing repeated sprints on a cycle ergometer it is common for the tester to require the athlete to maintain at least 60 rpm during the recovery period. Yet it is very often unreported whether this protocol factor was adhered to and thus, although seeking to standardize recovery, the potential insight for the efficacy of an athlete’s recovery process is lost.

Combat sports athletes require the ability for continual movement about the competitive arena for positioning an opponent and an ability for sustained upper-body isometric and dynamic contractions. While some of the repeat-effort data collected using common methods could elucidate this performance aspect, it is important to consider that sustained upper body isometric and dynamic contractions are not reflected easily in most repeat-effort testing as the methods used do not require significant strength, and therefore would not likely translate to combat sports. Previous research has tried to mitigate this issue with varying degrees of ecological validity by requiring athletes (judokas) to perform a series of unopposed throws of an opponent in their weight category between brief sprints (Franchini et al., 2011) or, alternatively, to push a weight sled (body mass relative) maximally for a specific distance (Barley et al., 2018b). However, these methods require further investigation to determine their applicability to their respective sports considering the aforementioned limitations of such protocols. Additionally, it is also important to consider if factors such as the level of competition or biological sex will influence the best practices for assessing physical capacities in combat athletes, which should be a topic of future research.

## Assessing Endurance in Combat Sports Athletes From a Physiological Perspective

Repeat-effort ability is maintained by a complicated relationship between anaerobic and aerobic metabolism, with the anaerobic system being mostly important in high-intensity performance and the aerobic system being important to recovery between efforts (Bishop et al., 2011; Girard et al., 2011). The initial high-intensity effort will be heavily reliant on anaerobic metabolism with increasing aerobic contribution as more efforts are completed (Girard et al., 2011). However, even in the final efforts of repeat-sprint protocols the majority of energy can still be yielded anaerobically, though to a lesser extent (Girard et al., 2011). A study by McGawley and Bishop (McGawley and Bishop, 2015) observed significant aerobic contribution in the final sprint of a 5 × 6-s maximal sprint protocol. As such, the high-intensity components of repeat-effort sports will be significantly influenced by an athlete’s anaerobic capacity even as the competition duration extends (Girard et al., 2011). Indeed, repeat-effort performance is likely to be heavily influenced by the accumulation of metabolic by-products, limitations in energy supply and neural fatigue (Girard et al., 2011). This is supported by studies that observe significant increases in blood metabolites during combat sports competitions (Bouhlel et al., 2006; Hanon et al., 2015) and the maximal cardiac response associated with combat sports competitions (Crisafulli et al., 2009; Hausen et al., 2017). As a result, common assessments of anaerobic capacity such as a Wingate or maximal accumulated oxygen deficit (MAOD) assessment will likely have important implications to combat sports endurance ability (Vandewalle et al., 1987; Faude et al., 2009; Bishop et al., 2011). The relationship between anaerobic capacity and competitive level in combat sports athletes has been observed in previous research (James et al., 2016b). However, the ability to recover from such high-intensity efforts during the low-intensity components will be driven primarily by the aerobic system to buffer hydrogen ion concentration and enhance phosphocreatine (PCr) regeneration (Balsom et al., 1992; Girard et al., 2011). This has been confirmed by previous research investigating the energy demands in taekwondo athletes during combat simulation (Campos et al., 2012). As such, greater aerobic fitness will likely improve repeat-effort ability in combat sports competitions by increasing oxygen availability, improving lactate removal and enhancing PCr regeneration (Tomlin and Wenger, 2001; Bishop et al., 2011). Increased aerobic fitness will also induce many physiological adaptations which could aid in combat sports endurance such as increased mitochondrial respiratory capacity, faster oxygen uptake kinetics, accelerated post-effort muscle re-oxygenation rate, improved lactate and ventilatory thresholds and a greater VO_2max_ (Bishop et al., 2011). However, it is important to remember that the relationship between maximal aerobic capacity and combat sports competitive level, or even repeat-effort performance in general does not appear to be linear (Bishop et al., 2011; Girard et al., 2011; Bridge et al., 2014; James et al., 2016b). We postulate that at the higher levels of combat sport competition there is a diminishing rate of return in the gross markers of aerobic capacity and adaptation. In fact, previous research has found sports-specific aerobic training in judo athletes to not improve VO_2max_ but to significantly affect ventilation thresholds, heart rate and VO_2_ recovery (Bonato et al., 2015). As such, other markers of aerobic fitness such as metabolic thresholds, economy, oxygen kinetics and the power output associated with VO_2max_ could more closely relate with fatigue development during repeat-effort exercise as observed in combat sports (Faude et al., 2009; Bishop et al., 2011). Due to the aforementioned differences between intermittent and continuous exercise, the ecological validity would increase if practitioners were to evaluate such factors during intermittent exercise protocols (Drust et al., 2000; Koralsztein and Billat, 2000) particularly with world class athletes. Further research is required to better understand exactly which markers of both anaerobic and aerobic fitness best relate to combat sports endurance.

## Conclusion

Combat sports are popular, physically demanding sports with a diverse competitive cohort around the globe. With a developing body of research, it is important to critically examine the current practices and how these may be best applied by the practitioner in the monitoring of athlete adaption process. Such a body evidence will not only help inform the assessment of endurance in combat sports athletes, but also the development of physical capacities which is a topic that needs further investigation. The current assessments of characteristics important for competitive combat sports performance, particularly those involved with endurance require further evaluation to determine their efficacy. This is important as many of the current methods may not be accurately assessing endurance ability or may lack the sensitivity to detect any changes. Endurance is a complicated characteristic comprised of many factors and as such cannot be comprehensively evaluated with a single test. A combination of assessments designed to simulate aspects of performance (in particular, repeat-effort ability) and others designed to better understand the underlying physiology will provide the most complete picture of combat sport endurance ability. However, while there is research investigating what physiological markers are important for combat sport athletes further research is needed to understand the relevant importance of variables such as metabolic thresholds and oxygen kinematics. When designing a test to simulate combat sport-specific endurance there are many things to consider, including how to induce a comparable physical and physiological load to competition alongside carefully choosing what activities will be included in the testing with respect to their ecological and scientific validity. Future research should investigate the potential impact that fatigue may have on combat sports-specific techniques and how such changes may influence assessments of endurance. Developing a better understanding the issues presented throughout this review will improve researchers’ ability to accurately assess characteristics relevant to combat sports performance, alongside allowing coaching staff to make appropriate training decisions and more effectively monitor the impact of such decisions.

## Author Contributions

OB and SG conceptualized the review topic and design. All other authors contributed to the crafting and editing of the manuscript.

## Conflict of Interest Statement

The authors declare that the research was conducted in the absence of any commercial or financial relationships that could be construed as a potential conflict of interest.
